# Supplementary material for: Screening of a Fraction with Higher Amyloid β Aggregation Inhibitory Activity from a Library Containing 210 Mushroom Extracts Using a Microliter-Scale High-Throughput Screening System with Quantum Dot Imaging
Source: Foods. 2024 Nov 22;13(23):3740. doi: 10.3390/foods13233740 (PMC11639962; doi:10.3390/foods13233740)
Supplement: Supplementary file 1 [file foods-13-03740-s001.zip › foods-3261517-supplementary.pdf]

Supplemental information

Screening of a Fraction with Higher Amyloid β Aggregation Inhibitory Activity from a Library Containing 210 Mushroom Extracts Using a Microliter-Scale High-Throughput Screening System with Quantum Dot Imaging

Gegentuya Huanood [1], Mahadeva M. M. Swamy [2], Rina Sasaki [1], Keiya Shimamori [1], Masahiro Kuragano [1], Enkhmaa Enkhbat [2], Yoshiko Suga [2], Masaki Anetai [2], Kenji Monde [2], Kiyotaka Tokuraku [1,*]

[1] Graduate School of Engineering, Muroran Institute of Technology, Muroran 050-8585, Japan
[2] Faculty of Advanced Life Science, Hokkaido University, Sapporo 001-0021, Japan
* Correspondence: tokuraku@mmm.muroran-it.ac.jp; Tel.: +81-0143-46-5721

Supplemental table S1: The names and classifications of 210 mushrooms from Hokkaido.
ND = not determined.

phylum	Class	Order	Scientific name	EC_{50} (μg/mL)
Ascomycota	Leotiomycetes	Helotiales	*Neobulgaria pura*	ND
Ascomycota	Leotiomycetes	Helotiales	*Bulgaria inquinans*	ND
Ascomycota	Pezizomycetes	pezizales	*Morchella conica*	ND
Ascomycota	Pezizomycetes	pezizales	*Peziza vesiculosa*	ND
Ascomycota	Pezizomycetes	pezizales	*Helvella crispa*	ND
Ascomycota	Pezizomycetes	pezizales	*Aleuria aurantia*	ND
Ascomycota	Sordariomycetes	Hypocreales	*Cordyceps sinensis*	ND
Ascomycota	Gepglossales	Geoglossales	*Spathularia flavida*	ND
Basidiomycota	Exobasidiomycetes	Tremellales	*Pseudohydnum gelatinosum*	ND
Basidiomycota	Agaricomycetes	Agaricales	*Rhodophyllus abortivus*	ND
Basidiomycota	Agaricomycetes	Agaricales	*Rhodophyllus sinuatus*	ND
Basidiomycota	Agaricomycetes	Agaricales	*Rhodophyllus rhodopolius*	ND
Basidiomycota	Agaricomycetes	Agaricales	*Pluteus atricapillus*	ND
Basidiomycota	Agaricomycetes	Agaricales	*Agrocybe praecox*	ND
Basidiomycota	Agaricomycetes	Agaricales	*Agrocybe farinacea*	ND
Basidiomycota	Agaricomycetes	Agaricales	*Lyophyllum decastes*	74.4
Basidiomycota	Agaricomycetes	Agaricales	*Laccaria amethystea*	ND
Basidiomycota	Agaricomycetes	Agaricales	*Collybia butyracea*	ND

Basidiomycota	Agaricomycetes	Agaricales	*Clitocybe clavipes*	ND
Basidiomycota	Agaricomycetes	Agaricales	*Lyophyllum connatum*	ND
Basidiomycota	Agaricomycetes	Agaricales	*Laccaria laccata*	ND
Basidiomycota	Agaricomycetes	Agaricales	*Tricholoma saponaceum*	ND
Basidiomycota	Agaricomycetes	Agaricales	*Tricholoma sejunctum*	ND
Basidiomycota	Agaricomycetes	Agaricales	*Tricholoma robustum*	ND
Basidiomycota	Agaricomycetes	Agaricales	*Armillariella mellea*	ND
Basidiomycota	Agaricomycetes	Agaricales	*Leucopaxillus giganteus*	ND
Basidiomycota	Agaricomycetes	Agaricales	*Tricholoma ustale*	ND
Basidiomycota	Agaricomycetes	Agaricales	*Clitocybe nebularis*	ND
Basidiomycota	Agaricomycetes	Agaricales	*Lepista nuda*	ND
Basidiomycota	Agaricomycetes	Agaricales	*Tricholoma albobrunneum*	ND
Basidiomycota	Agaricomycetes	Agaricales	*Tricholoma flavovirens*	ND
Basidiomycota	Agaricomycetes	Agaricales	*Clitocybe robusta*	ND
Basidiomycota	Agaricomycetes	Agaricales	*Lyophyllum fumosum*	ND
Basidiomycota	Agaricomycetes	Agaricales	*Tricholoma album*	ND
Basidiomycota	Agaricomycetes	Agaricales	*Lepista irina*	ND
Basidiomycota	Agaricomycetes	Agaricales	*Tricholoma virgatum*	ND
Basidiomycota	Agaricomycetes	Agaricales	*Flammulina velutipes*	ND
Basidiomycota	Agaricomycetes	Agaricales	*Panellus serotinus*	ND
Basidiomycota	Agaricomycetes	Agaricales	*Oudemansiella mucida*	ND
Basidiomycota	Agaricomycetes	Agaricales	*Marasmius maximus*	ND
Basidiomycota	Agaricomycetes	Agaricales	*Tricholomopsis rutilans*	ND
Basidiomycota	Agaricomycetes	Agaricales	*Collybia acervata*	ND
Basidiomycota	Agaricomycetes	Agaricales	*Clitocybe inornata*	ND
Basidiomycota	Agaricomycetes	Agaricales	*Mycena pura*	ND
Basidiomycota	Agaricomycetes	Agaricales	*Clitocybe lignatilis*	ND
Basidiomycota	Agaricomycetes	Agaricales	*Lepista sordida*	ND
Basidiomycota	Agaricomycetes	Agaricales	*Rhodocollybia maculata*	ND
Basidiomycota	Agaricomycetes	Agaricales	*Oudemansiella brunneomaginata*	ND
Basidiomycota	Agaricomycetes	Agaricales	*Lampteromyces japonicus*	ND
Basidiomycota	Agaricomycetes	Agaricales	*Laccaria laccata*	ND
Basidiomycota	Agaricomycetes	Agaricales	*Clitocybe gibba*	ND
Basidiomycota	Agaricomycetes	Agaricales	*Lyophyllum shimeji*	ND
Basidiomycota	Agaricomycetes	Agaricales	*Clitocybula esculenta*	ND
Basidiomycota	Agaricomycetes	Agaricales	*Pseudoclitocybe cyathiformis*	ND
Basidiomycota	Agaricomycetes	Agaricales	*Tricholoma*	ND

			portentosum	
Basidiomycota	Agaricomycetes	Agaricales	*Hypsizigus marmoreus*	ND
Basidiomycota	Agaricomycetes	Agaricales	*Pleurocybella porrigens*	ND
Basidiomycota	Agaricomycetes	Agaricales	*Amanita subjunquillea*	55.7
Basidiomycota	Agaricomycetes	Agaricales	*Amanita hemibapha*	ND
Basidiomycota	Agaricomycetes	Agaricales	*Amanita pantherina*	ND
Basidiomycota	Agaricomycetes	Agaricales	*Amanita muscaria*	ND
Basidiomycota	Agaricomycetes	Agaricales	*Amanita Imazekii*	ND
Basidiomycota	Agaricomycetes	Agaricales	*Amanita rubescens*	ND
Basidiomycota	Agaricomycetes	Agaricales	*Amanita citrina* var. *citrina*	ND
Basidiomycota	Agaricomycetes	Agaricales	*Amanita ibotengutake*	ND
Basidiomycota	Agaricomycetes	Agaricales	*Hygrophorus russula*	ND
Basidiomycota	Agaricomycetes	Agaricales	*Hygrophorus calophyllus*	ND
Basidiomycota	Agaricomycetes	Agaricales	*Hygrophorus chrysodon*	ND
Basidiomycota	Agaricomycetes	Agaricales	*Hygrophorus camarophyllus*	ND
Basidiomycota	Agaricomycetes	Agaricales	*Hygrophorus lucorum*	ND
Basidiomycota	Agaricomycetes	Agaricales	*Hygrophorus eburneus*	ND
Basidiomycota	Agaricomycetes	Agaricales	*Hygrophorus leucophaeus*	ND
Basidiomycota	Agaricomycetes	Agaricales	*Hygrophorus pudorinus*	ND
Basidiomycota	Agaricomycetes	Agaricales	*Camarophyllus virgineus*	ND
Basidiomycota	Agaricomycetes	Agaricales	*Armillaria nigrescens* Kawam.	ND
Basidiomycota	Agaricomycetes	Agaricales	*Camarophyllus pratensis*	ND
Basidiomycota	Agaricomycetes	Agaricales	*Macrolepiota procera*	ND
Basidiomycota	Agaricomycetes	Agaricales	*Leucoagaricus naucinus*	ND
Basidiomycota	Agaricomycetes	Agaricales	*Agaricus abruptibulbus*	ND
Basidiomycota	Agaricomycetes	Agaricales	*Phaeolepiota aurea*	ND
Basidiomycota	Agaricomycetes	Agaricales	*Agaricus campestris*	ND
Basidiomycota	Agaricomycetes	Agaricales	*Cystoderma amianthinum*	ND
Basidiomycota	Agaricomycetes	Agaricales	*Agaricus subperonatus*	ND
Basidiomycota	Agaricomycetes	Agaricales	*Psathyrella candolliana*	ND
Basidiomycota	Agaricomycetes	Agaricales	*Coprinus comatus*	ND
Basidiomycota	Agaricomycetes	Agaricales	*Psathyrella velutina*	ND
Basidiomycota	Agaricomycetes	Agaricales	*Psathyrella multissima*	ND
Basidiomycota	Agaricomycetes	Agaricales	*Pleurotus ostreatus*	ND

Basidiomycota	Agaricomycetes	Agaricales	*Pleurotus pulmonarius*	ND
Basidiomycota	Agaricomycetes	Agaricales	*Schizophyllum commune*	ND
Basidiomycota	Agaricomycetes	Agaricales	*Pleurotus cornucopiae* var. *citrinopileatus*	ND
Basidiomycota	Agaricomycetes	Agaricales	*Lentinellus ursinus*	ND
Basidiomycota	Agaricomycetes	Agaricales	*Lentinellus cochleatus*	ND
Basidiomycota	Agaricomycetes	Agaricales	*Lentinus edodes*	ND
Basidiomycota	Agaricomycetes	Agaricales	*Cortinarius crocolitus*	ND
Basidiomycota	Agaricomycetes	Agaricales	*Cortinarius vibratilis*	ND
Basidiomycota	Agaricomycetes	Agaricales	*Cortinarius triumphans*	ND
Basidiomycota	Agaricomycetes	Agaricales	*Cortinarius purpurascens*	ND
Basidiomycota	Agaricomycetes	Agaricales	*Cortinarius aureobrunneus*	ND
Basidiomycota	Agaricomycetes	Agaricales	*Cortinarius trivialis*	ND
Basidiomycota	Agaricomycetes	Agaricales	*Descolea flavoannulata*	ND
Basidiomycota	Agaricomycetes	Agaricales	*Inocybe fastigiata*	ND
Basidiomycota	Agaricomycetes	Agaricales	*Inocybe umbratica*	ND
Basidiomycota	Agaricomycetes	Agaricales	*Dermocybe cinnamomea*	ND
Basidiomycota	Agaricomycetes	Agaricales	*Gymnopilus liquiritiae*	ND
Basidiomycota	Agaricomycetes	Agaricales	*Cortinarius allutus*	ND
Basidiomycota	Agaricomycetes	Agaricales	*Cortinarius azureus*	ND
Basidiomycota	Agaricomycetes	Agaricales	*Cortinarius xanthophyllus*	ND
Basidiomycota	Agaricomycetes	Agaricales	*Cortinarius duracinus*	ND
Basidiomycota	Agaricomycetes	Agaricales	*Cortinarius purpurascens*	ND
Basidiomycota	Agaricomycetes	Agaricales	*Russula delica var. glaucophylla*	ND
Basidiomycota	Agaricomycetes	Agaricales	*Lactarius torminosus*	ND
Basidiomycota	Agaricomycetes	Agaricales	*Lactarius flavidulus*	ND
Basidiomycota	Agaricomycetes	Agaricales	*Lactarius laeticolorus*	ND
Basidiomycota	Agaricomycetes	Agaricales	*Russula sanguinea*	ND
Basidiomycota	Agaricomycetes	Agaricales	*Lactarius akahatsu*	ND
Basidiomycota	Agaricomycetes	Agaricales	*Russula nigricans*	ND
Basidiomycota	Agaricomycetes	Agaricales	*Lactarius piperatus*	ND
Basidiomycota	Agaricomycetes	Agaricales	*Lactarius necator*	ND
Basidiomycota	Agaricomycetes	Agaricales	*Lactarius scrobiculatus*	ND
Basidiomycota	Agaricomycetes	Agaricales	*Russula emetica*	ND
Basidiomycota	Agaricomycetes	Agaricales	*Lactarius chrysorrheus*	ND
Basidiomycota	Agaricomycetes	Agaricales	*Russula cyanoxantha*	ND

Basidiomycota	Agaricomycetes	Agaricales	*Russula senecis*	ND
Basidiomycota	Agaricomycetes	Agaricales	*Russula foetens*	ND
Basidiomycota	Agaricomycetes	Agaricales	*Lactarius volemus*	ND
Basidiomycota	Agaricomycetes	Agaricales	*Lactarius uvidus*	ND
Basidiomycota	Agaricomycetes	Agaricales	*Russula mariae*	ND
Basidiomycota	Agaricomycetes	Agaricales	*Lactarius hysginus*	ND
Basidiomycota	Agaricomycetes	Agaricales	*Lycoperdon perlatum*	ND
Basidiomycota	Agaricomycetes	Agaricales	*Calvatia craniiformis*	ND
Basidiomycota	Agaricomycetes	Agaricales	*Pholiota flammans*	15.8
Basidiomycota	Agaricomycetes	Agaricales	*Pholiota aurivella*	47.7
Basidiomycota	Agaricomycetes	Agaricales	*Naematoloma sublateritium*	ND
Basidiomycota	Agaricomycetes	Agaricales	*Pholiota terrestris*	ND
Basidiomycota	Agaricomycetes	Agaricales	*Pholiota nameko*	ND
Basidiomycota	Agaricomycetes	Agaricales	*Pholiota squarrosa*	ND
Basidiomycota	Agaricomycetes	Agaricales	*Naematoloma fasciculare*	ND
Basidiomycota	Agaricomycetes	Agaricales	*Naematoloma gracile*	ND
Basidiomycota	Agaricomycetes	Agaricales	*Pholiota spumosa*	ND
Basidiomycota	Agaricomycetes	Agaricales	*Stropharia rugosoannulata*	ND
Basidiomycota	Agaricomycetes	Agaricales	*Pholiota albocrenulata*	ND
Basidiomycota	Agaricomycetes	Agaricales	*Pholiota squarrosoides*	ND
Basidiomycota	Agaricomycetes	Agaricales	*Stropharia aeruginosa*	ND
Basidiomycota	Agaricomycetes	Agaricales	*Pholiota lubrica*	ND
Basidiomycota	Agaricomycetes	Agaricales	*Pholiota lenta*	ND
Basidiomycota	Agaricomycetes	Agaricales	*Naematoloma capnoides*	ND
Basidiomycota	Agaricomycetes	Agaricales	*Pholiota adiposa*	ND
Basidiomycota	Agaricomycetes	Boletales	*Suillus grevillei*	ND
Basidiomycota	Agaricomycetes	Boletales	*Leccinum versipelle*	ND
Basidiomycota	Agaricomycetes	Boletales	*Leccinum scabrum*	ND
Basidiomycota	Agaricomycetes	Boletales	*Suillus granulatus*	ND
Basidiomycota	Agaricomycetes	Boletales	*Boletinus cavipes*	ND
Basidiomycota	Agaricomycetes	Boletales	*Suillus americanus*	ND
Basidiomycota	Agaricomycetes	Boletales	*Boletus edulis*	ND
Basidiomycota	Agaricomycetes	Boletales	*Tylopilus fumosipes*	ND
Basidiomycota	Agaricomycetes	Boletales	*Boletus fraternus*	ND
Basidiomycota	Agaricomycetes	Boletales	*Boletus subvelutipes*	ND
Basidiomycota	Agaricomycetes	Boletales	*Phylloporus bellus*	ND
Basidiomycota	Agaricomycetes	Boletales	*Tylopilus vinosobrunneus*	ND
Basidiomycota	Agaricomycetes	Boletales	*Boletus aereus*	ND

Basidiomycota	Agaricomycetes	Boletales	*Tylopilus neofelleus*	ND
Basidiomycota	Agaricomycetes	Boletales	*Boletus venenatus*	ND
Basidiomycota	Agaricomycetes	Boletales	*Gyrodon lividus*	ND
Basidiomycota	Agaricomycetes	Boletales	*Suillus laricinus*	ND
Basidiomycota	Agaricomycetes	Boletales	*Leccinum holopus*	ND
Basidiomycota	Agaricomycetes	Boletales	*Boletus reticulatus*	ND
Basidiomycota	Agaricomycetes	Boletales	*Strobilomyces cofusus*	ND
Basidiomycota	Agaricomycetes	Boletales	*Pisolithus tinctorius*	7.15
Basidiomycota	Agaricomycetes	Boletales	*Paxillus involutus*	ND
Basidiomycota	Agaricomycetes	Geastrales	*Geastrum triplex*	ND
Basidiomycota	Agaricomycetes	Gomphales	*Ramaria formosa*	ND
Basidiomycota	Agaricomycetes	Gomphales	*Ramaria flava*	ND
Basidiomycota	Agaricomycetes	Phallales	*Phallus impudicus*	ND
Basidiomycota	Agaricomycetes	Auriculariales	*Auricularia auricula*	ND
Basidiomycota	Agaricomycetes	cantharellales	*Cantharellus cibarius*	ND
Basidiomycota	Agaricomycetes	cantharellales	*Mycoleptodonoides aitchisonii*	ND
Basidiomycota	Agaricomycetes	Hymenochaetales	*Phellinus igniarius*	7.54
Basidiomycota	Agaricomycetes	Hymenochaetales	*Fuscoporia obliqua*	7.94
Basidiomycota	Agaricomycetes	Hymenochaetales	*Onnia scaura*	10.4
Basidiomycota	Agaricomycetes	Polyporales	*Polyporus squamosus*	ND
Basidiomycota	Agaricomycetes	Polyporales	*Fomes fomentarius*	17.2
Basidiomycota	Agaricomycetes	Polyporales	*Tyromyces sambuceus*	53.5
Basidiomycota	Agaricomycetes	Polyporales	*Fomitopsis pinicola*	58.0
Basidiomycota	Agaricomycetes	Polyporales	*Heterobasidion insularis*	77.1
Basidiomycota	Agaricomycetes	Polyporales	*Grifola frondosa*	85.2
Basidiomycota	Agaricomycetes	Polyporales	*Polyporellus badius*	ND
Basidiomycota	Agaricomycetes	Polyporales	*Polyporellus brumalis*	ND
Basidiomycota	Agaricomycetes	Polyporales	*Piptoporus betulinus*	ND
Basidiomycota	Agaricomycetes	Polyporales	*Daedalea dickinsii*	ND
Basidiomycota	Agaricomycetes	Polyporales	*Bondarzewia montana*	ND
Basidiomycota	Agaricomycetes	Polyporales	*Coriolus versicolor*	ND
Basidiomycota	Agaricomycetes	Polyporales	*Tyromyces spumens*	ND
Basidiomycota	Agaricomycetes	Polyporales	*Polyporus alveolaris*	ND
Basidiomycota	Agaricomycetes	Polyporales	*Oligoporus tephroleucus*	19.2
Basidiomycota	Agaricomycetes	Polyporales	*Laetiporus sulphureus* var. *miniatus*	ND
Basidiomycota	Agaricomycetes	Polyporales	*Sparassis crispa*	ND
Basidiomycota	Agaricomycetes	Aphyllophorales	*Polyporus umbellatus* Fries	ND
Basidiomycota	Agaricomycetes	Aphyllophorales	*Poria cocos* Wolf	ND

Basidiomycota	Agaricomycetes	Aphyllophorales	*Elfvingia applanata*	18.1
			Elfvingia applanata (Purchased)	6.21
Basidiomycota	Agaricomycetes	Aphyllophorales	*Ganoderma lucidum*	63.3
Basidiomycota	Agaricomycetes	Russulales	*Albatrellus dispansus*	71.9
Basidiomycota	Agaricomycetes	Russulales	*Albatrellus caeruleoporus*	ND
Basidiomycota	Agaricomycetes	Thelephorales	*Boletopsis leucomelas*	37.5
Basidiomycota	Agaricomycetes	Thelephorales	*Thelephora palmata*	81.1
Basidiomycota	Agaricomycetes	Thelephorales	*Sarcodon scabrosus*	ND
Basidiomycota	Agaricomycetes	Thelephorales	*Sarcodon leucopus*	ND
Basidiomycota	Agaricomycetes	Thelephorales	*Sarcodon aspratus*	ND
Basidiomycota	Agaricomycetes	Thelephorales	*Hydnellum caeruleum*	ND
Basidiomycota	Agaricomycetes	Thelephorales	*Hydnellum concrescens*	ND

A

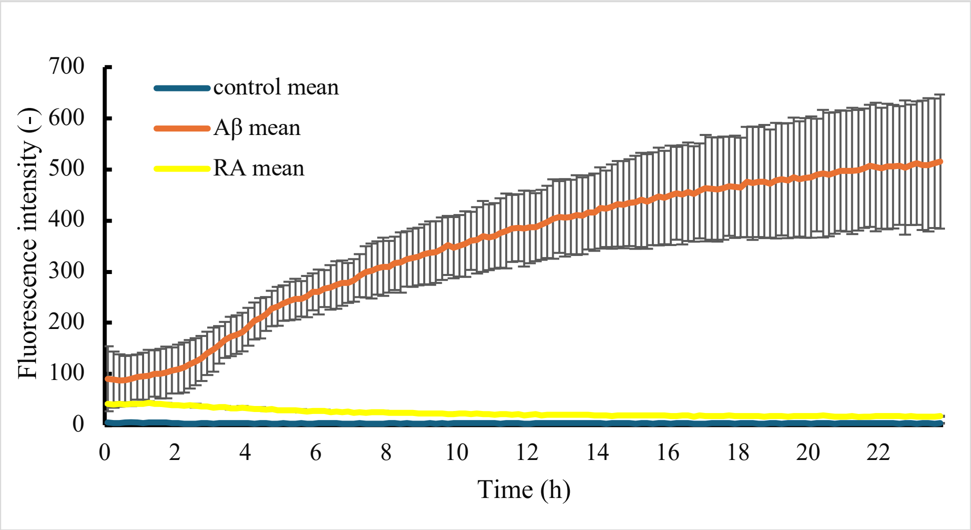
700
600
500
400
300
200
100
0
Fluorescence intensity (-)
control mean
Aβ mean
RA mean
0
2
4
6
8
10
12
14
16
18
20
22
Time (h)

B

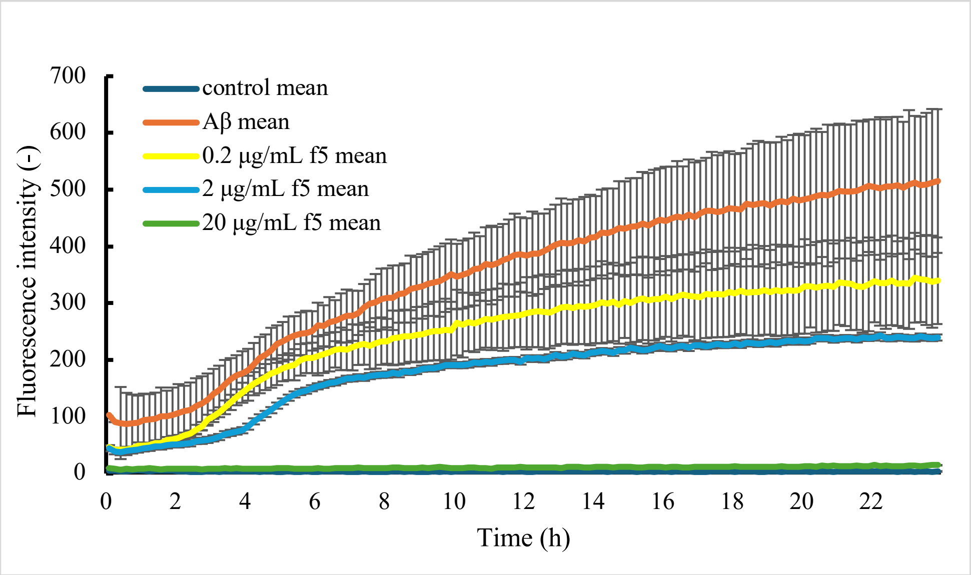
700
600
500
400
300
200
100
0
Fluorescence intensity (-)
control mean
Aβ mean
0.2 μg/mL f5 mean
2 μg/mL f5 mean
20 μg/mL f5 mean
0
2
4
6
8
10
12
14
16
18
20
22
Time (h)

C

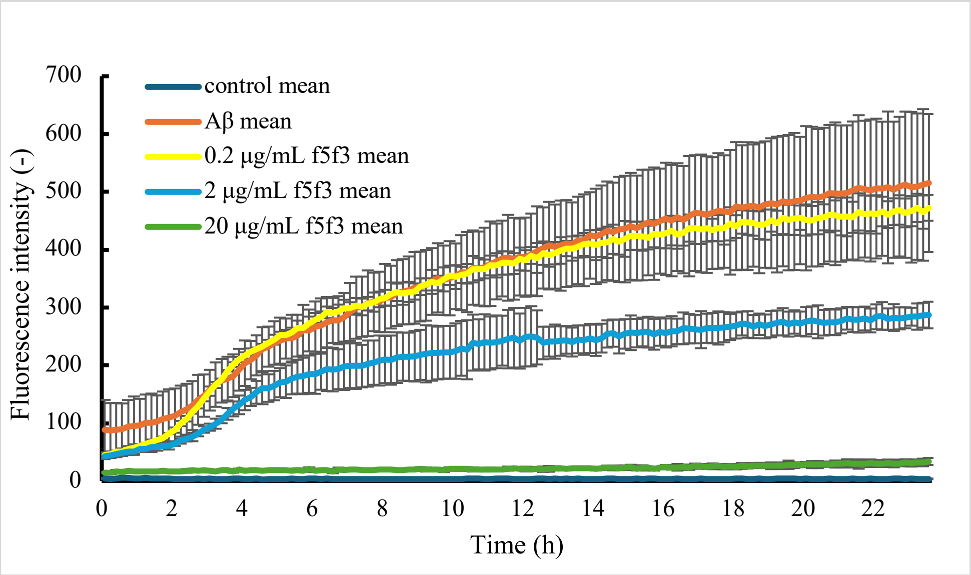
700
600
500
400
300
200
100
0
Fluorescence intensity (-)
control mean
Aβ mean
0.2 μg/mL f5f3 mean
2 μg/mL f5f3 mean
20 μg/mL f5f3 mean
0
2
4
6
8
10
12
14
16
18
20
22
Time (h)

Supplemental figure S1. The aggregation inhibition activity of three inhibitors (RA, f5, f5f3) against Aβ was evaluated by the ThT assay. (A) Control: 5% DMSO + 1 × PBS + ThT solution; Aβ: Aβ solution + ThT solution; RA: RA solution + Aβ solution + ThT solution. (B) Control: 5% DMSO + 1 × PBS + ThT solution; Aβ: Aβ solution + ThT solution; f5: f5 solution (0.2, 2 and 20 μg/mL) + Aβ solution + ThT solution. (C) Control: 5% DMSO + 1 × PBS + ThT solution; Aβ: Aβ solution + ThT solution; f5f3: f5f3 solution (0.2, 2 and 20 μg/mL) + Aβ solution + ThT solution. Final concentration (Aβ: 25 μM; RA: 300 μM; ThT: 20 μM). (n = 3). The Microplate Reader (SH-9000, Yamato, Tokyo, Japan) employed the following settings: 450 nm excitation wavelength, 490 nm emission wavelength.

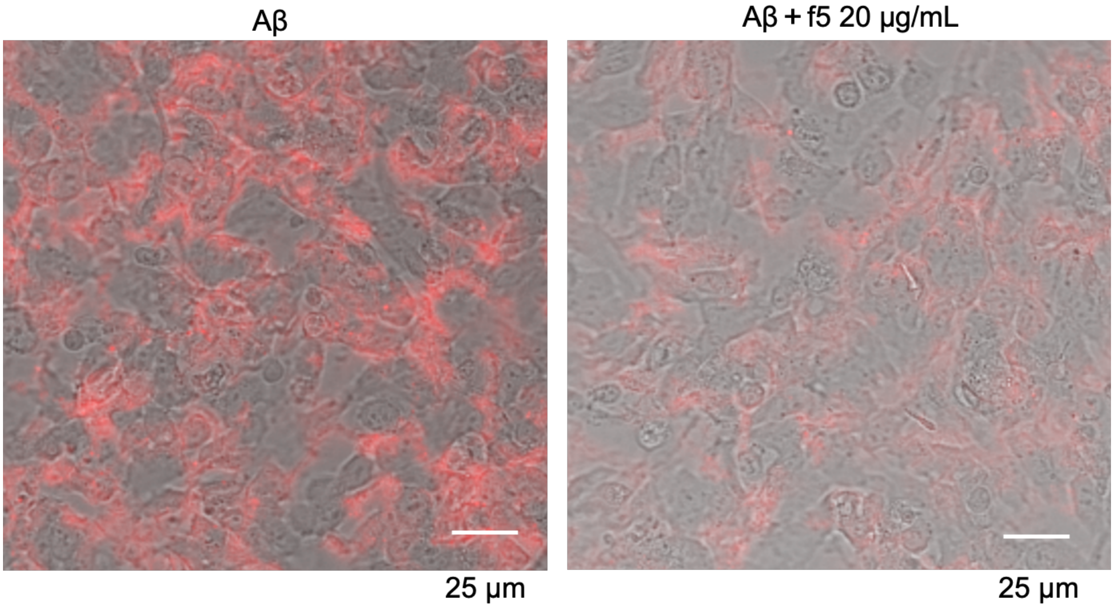

Supplemental figure S2. Merge images of the fraction f5 of t037 on extracellular Aβ deposition in SH-SY5Y cells. The left one is the merge image of the Aβ group, and the right one is the merge image of the Aβ plus f5 20 μg/mL group.
